# Nursing‐Led Knowledge Translation Strategies to Improve Patient Safety in Hospital Settings: A Scoping Review

**DOI:** 10.1111/jan.70621

**Published:** 2026-04-17

**Authors:** Jhenyfer Amanda Ciriaco Canhete, Vânia Ana Silveira Muniz, Milene Negri Reiser, Chantal Backman, Gabriela Marcellino de Melo Lanzoni, Ana Lúcia Schaefer Ferreira de Mello

**Affiliations:** ^1^ Graduate Program in Nursing Federal University of Santa Catarina Florianópolis Santa Catarina Brazil; ^2^ School of Nursing, Faculty of Health Sciences University of Ottawa Ottawa Ontario Canada

## Abstract

**Aim:**

To conduct a scoping review of nurse‐led Knowledge Translation strategies aimed at promoting and enhancing patient safety in hospital settings.

**Design:**

Scoping review.

**Methods:**

This review followed the Joanna Briggs Institute methodology and was reported according to PRISMA‐ScR.

**Data Sources:**

Twelve electronic databases and additional grey literature sources were searched for studies published between 2002 and 2023, with no language restrictions.

**Results:**

From 23,691 records identified, 59 studies were included. The majority (*n* = 56) employed multifaceted Knowledge Translation strategies, incorporating simulation, audits, digital tools and interprofessional education. The interventions focused on patient safety‐related events, including falls, pressure injuries and catheter‐associated complications. Nursing leadership emerged as a key component, particularly in team training, developing care protocols and delivering feedback. Outcomes included reductions in adverse events, improved adherence to clinical guidelines and cost savings. Yet, sustaining behaviour changes over time and limited interprofessional and family engagement remained recurrent challenges.

**Conclusions:**

Nurse‐led Knowledge Translation strategies were heterogeneous, with increasing use of simulations, technologies and multifaceted approaches. Evidence suggests potential associations with fewer adverse events, improved care quality, individualized planning and cost efficiency. Challenges related to the sustainability of interventions persist. Findings underscore the importance of investing in nursing leadership and capacity‐building to strengthen patient safety.

**Implications for the Profession and/or Patient Care:**

Strengthening nurse‐led KT capacities may enhance evidence‐based care and improve safety outcomes. Investment in leadership and tailored implementation is critical.

**Impact:**

What problem did the study address? The limited synthesis of how nurses lead KT strategies to improve patient safety in hospitals. What were the main findings? Most strategies were multifaceted, context‐sensitive and associated with improved care processes and safety indicators. Where and on whom will the research have an impact? Findings are relevant to hospital nurses, nurse educators, managers and health systems seeking to implement evidence‐informed safety interventions.

**Reporting Method:**

This scoping review followed the PRISMA‐ScR reporting guideline.

**Patient or Public Contribution:**

This study did not include patient or public involvement in its design, conduct or reporting.

**Trial Registration:**

Open Science Framework (OSF); registration identifier: 10.17605/OSF.IO/K3VJC

## What Does This Paper Contribute to the Wider Global Clinical Community?

- Provides a comprehensive synthesis of nurse‐led Knowledge Translation strategies to improve patient safety in hospital settings.
- Highlights the predominance of multifaceted interventions and the central role of nursing leadership in implementation
- Identifies key challenges to sustainability and offers insights to inform practice, policy and future implementation research.

## 1 Introduction

Knowledge Translation (KT) is understood as the dynamic and iterative process that integrates evidence into practice through the synthesis, dissemination, exchange and ethical application of knowledge to improve health outcomes and strengthen health systems (Canadian Institutes of Health Research 2016).

Building on this perspective, KT represents an essential mechanism for narrowing the well‐recognized gap between what is known from research and what is done in clinical practice (Graham et al. 2006). However, challenges persist in implementing and sustaining the use of evidence in healthcare policies and practices. Studies suggest that, on average, only 60% of healthcare practices align with evidence‐based guidelines (Braithwaite et al. 2018; Runciman et al. 2012).

Patient Safety is a fundamental pillar of high‐quality healthcare, defined as minimizing unnecessary harm to patients to an acceptable level (Runciman et al. 2009). Despite advancements in clinical research, a significant gap remains between the generation of knowledge demonstrating the benefits of new treatment interventions and their consistent implementation in routine clinical practice. This gap has a direct impact on patient safety and the quality of care (Needham 2010).

Addressing this issue requires targeted KT strategies that facilitate the translation of research findings into practice, benefiting patients, families, healthcare professionals, managers and organizations (Flenady et al. 2022). Although KT shares theoretical and methodological foundations with the broader field of Implementation Science, it emphasizes the practical, context‐sensitive and collaborative processes that are central to nursing practice, serving as a pragmatic guiding concept for analysing efforts to improve patient safety (Lundkvist et al. 2021).

In hospital settings, nursing professionals face daily challenges that require quick, safe and up‐to‐date decision‐making. Solid research evidence plays a crucial role in supporting, questioning and enhancing nursing education, care delivery and management (Descatha and Fadel 2023). The translation of research evidence into clinical practice is essential for ensuring safe, transparent, effective and efficient healthcare while also meeting patient expectations (Rabelo‐Silva et al. 2022).

Through Knowledge Translation (KT), nursing can expand its knowledge base and develop evidence‐based actions, including diagnostic classifications, outcome predictions and tailored patient‐centred interventions (Chisholm et al. 2025; Rabelo‐Silva et al. 2022; Wallin 2009). A recent study further demonstrated that collaborative and context‐sensitive KT approaches can lead to superior outcomes compared with isolated disciplinary strategies, particularly when multifaceted interventions are adapted to the specific realities and needs of healthcare teams (Graverholt et al. 2024). Despite the growing integration of KT into nursing practice, more rigorous study designs are still needed to optimize its application and generate tangible benefits for healthcare teams, patients and families.

Although research on KT has advanced, significant global disparities persist. Researchers in low‐ and middle‐income countries (LMICs) rarely conduct KT initiatives and face multilevel barriers that limit their ability to implement evidence‐based practices. Among the main obstacles are insufficient KT knowledge and skills, particularly regarding the communication of research findings, as well as limited funding and the absence of institutional guidelines, structures and incentives to support KT practice. As a result, the evidence based on effective interventions to strengthen KT in these countries remains scarce and of limited quality (Kalbarczyk et al. 2021).

Given that the sustainability of KT interventions is a recognized challenge (Chicoine and Straus 2025), healthcare professionals and organizations must leverage their influence to disseminate evidence and enhance patient safety. However, existing evidence on the implementation of KT strategies in nursing and their impact on patient safety remains limited, and it does not explicitly focus on nursing interventions or patient safety in hospital settings (Fontaine et al. 2024). Considering these gaps, the focus of this scoping review was to explore the literature on nursing‐led KT strategies to improve patient safety in hospital environments. The aim was to provide valuable insights for researchers, health policymakers and service providers.

## 2 The Review

### 2.1 Aim

To conduct a scoping review of nurse‐led Knowledge Translation strategies aimed at promoting and enhancing patient safety in hospital settings.

## 3 Method

### 3.1 Design

This scoping review was conducted in accordance with the methodology proposed by the Joanna Briggs Institute (JBI) (Peters et al. 2020) and reported following the Preferred Reporting Items for Systematic reviews and Meta‐Analyses extension for Scoping Reviews (PRISMA‐ScR) (Tricco et al. 2018). The protocol was registered in advance on the Open Science Framework on October 19, 2023. The central question guiding this review is: What Knowledge Translation strategies do nurses implement to promote and improve patient safety in hospital settings, and how do these strategies impact patients, healthcare professionals, managers and healthcare organizations?

Although implementation is often treated as a separate field, this review considered it a core element of KT strategies, particularly those aimed at incorporating evidence‐based interventions into nursing practice (Graham et al. 2006). In this review, and consistent with interpretations in KT literature (Straus et al. 2009; Wallin 2009), implementation was treated as the operational phase of KT, that is, the stage through which evidence becomes embedded in clinical practice, rather than as a distinct or competing concept. Consequently, strategies traditionally labelled as ‘implementation strategies’ were included only when they formed part of a nursing‐driven KT process focused on embedding evidence into routine patient safety practices. This approach ensured conceptual consistency and allowed the review to capture how nurses operationalize KT to promote safer clinical care.

### 3.2 Search Methods

The lead researcher, supported by a research librarian, developed the literature search on October 16, 2023. The databases searched included the Cumulative Index to Nursing and Allied Health Literature (CINAHL/EBSCO), EMBASE (Elsevier), BIREME/LILACS (Latin American and Caribbean Health Sciences Information System), PubMed/MEDLINE, Scientific Electronic Library Online (SciELO), Scopus (Elsevier), Web of Science (Clarivate Analytics), ProQuest Dissertations and Theses Global (PQDT Global), Google Scholar, Cochrane Library, LILACS/BDENF, Open Access Theses and Dissertations (OATD) and the CAPES Catalogue of Theses and Dissertations. Although the search strategy was developed and executed on October 16, 2023, records were exported on November 8, 2023. This timing led to slight variations in the number of retrieved studies due to ongoing database updates.

The Population, Concept, Context (PCC) framework guided the identification of keywords and synonyms associated with the research question. The specific elements and their corresponding keywords and synonyms are presented in Table 1. The search strategy, including all identified keywords and index terms, was adapted for each database and/or information source. The search strings were generated by combining keywords and synonyms using Boolean operators and modifiers (File S1).

**TABLE 1:** Populations, concept and context (PCC) elements.

PCC element	Definition		MeSH terms, keyword and synonyms
Population	Nurses and nursing staff involved in designing, implementing or evaluating nurse‐led Knowledge Translation (KT) strategies in hospital settings.		Nurses and nursing staff
Concept	Literature describing nursing‐led knowledge translation strategies in practice for patient safety (PS) and their impacts, in a broader approach.	Knowledge Translation strategies (actions and processes) are effectively implemented Report of impacts: measures of outcomes, effectiveness and sustainability of knowledge‐to‐practice translation.	Knowledge translation Knowledge transfer Knowledge brokering Knowledge management Knowledge translation strategies Evidence‐based practice interventions Knowledge sharing Integrated knowledge translation Knowledge translation interventions Knowledge Integration Translational Research Evidence‐Based Practice Knowledge Application Research Utilization Knowledge Exchange Knowledge Dissemination Knowledge Mobilization
		Patient Safety is considered the prevention/reduction of risk of unnecessary harm associated with healthcare.	Patient Safety Safety Patient Safeties Safeties Patient Safety Management Safety culture Culture of safety Safety climate Quality Care quality Quality of care Care quality outcomes Patient Harm Patient outcome Adverse event Harmful incident Healthcare‐associated harm, Hazard, Reportable circumstance, Near miss, No harm incident, Adverse reaction, Side effect, Preventable, Patient safety incident Harm Disease Injury Suffering Disability Degree of harm Error recovery Violation Risk Actions taken to reduce risk System failure System improvement Resilience
Context	Hospital setting		Hospital and its units Hospital Unit

### 3.3 Inclusion and Exclusion Criteria

The articles found were assessed for eligibility using the Population–Concept–Context (PCC) framework (Table 1).

Population: Nurses and nursing staff involved in the design, implementation or evaluation of Knowledge Translation (KT) strategies in hospital settings. Although the review did not target a specific patient population, the focus on nursing professionals reflects the aim to explore nursing‐led KT initiatives rather than patient‐level outcomes.

Concept: Literature describing nursing‐led knowledge translation strategies in practice for patient safety (PS) and their impacts in a broader approach.

Context: Studies conducted in hospital settings, regardless of country or type of clinical unit.

Studies had to be conducted in hospital settings, with no restrictions on publication date or language, to allow for a broad and temporal exploration of the use of Knowledge Translation strategies across different contexts. Eligibility was also extended to studies addressing related concepts, such as knowledge utilization, knowledge brokering and knowledge transfer, when linked to patient safety outcomes.

The identification of nurse‐led strategies was based on explicit statements within the included studies indicating that nurses led the intervention. In cases where authorship or leadership was unclear, the determination was made by verifying the academic background and professional credentials of the authors responsible for the intervention.

Studies that did not mention nursing practices, actions, roles, responsibilities or interventions led by nurses, performed by nursing professionals or oriented by nursing leadership or management were excluded. Studies that did not specifically address Knowledge Translation strategies applied to patient safety were also excluded.

#### 3.3.1 Inclusion

Peer‐reviewed journal articles, academic theses, dissertations, technical reports and institutional documents describing nursing‐led KT strategies aimed at improving patient safety in hospital settings.

Studies are conducted in hospital settings regardless of country or clinical unit type.

No restrictions on publication date or language.

Studies addressing knowledge utilization, brokering or transfer linked to patient safety outcomes.

Explicit indication of nurse leadership in the intervention or deduced via authorship credentials.

#### 3.3.2 Exclusion

Studies without explicit mention of nursing‐led practices, roles or interventions.

Studies not addressing Knowledge Translation strategies applied to patient safety.

### 3.4 Search Outcome

The search results were exported to Covidence, an online platform for systematic review management, where duplicates were removed and studies were screened.

An external expert in scoping reviews supervised the entire selection and analysis process. An initial screening of titles and abstracts was done according to the inclusion and exclusion criteria. Five trained reviewers (all co‐authors) independently selected the studies. Studies were included if they received two votes in favour. Full texts eligible for inclusion were read, independently analysed and examined by the same five reviewers.

At the full‐text screening stage, the same inclusion criteria were applied. In cases of disagreement, a third researcher was involved in the final decision. The reasons for excluding the full texts were recorded to track the decisions made and are provided in File S2. A total of 23,691 records were identified across databases. After removing duplicates, 59 studies met the eligibility requirements and were included in the review.

### 3.5 Quality Appraisal

The quality appraisal of the studies was performed using the Quality Assessment for Diverse Studies (QuADS) tool (Harrison et al. 2021). It is a general tool for methodological and reporting quality assessment, designed for use in systematic reviews that include mixed‐methods studies or multiple study designs. After applying the 13 QuADS criteria (maximum score of 39), final scores are presented in a File S3. Based on the results from the QuADS tool, the overall methodological quality of the included studies was generally high, although some variations were observed. A significant number of studies demonstrated strong quality, with several achieving perfect or near‐perfect scores, such as Blanco‐Mavillard et al. (2021), Choong et al. (2021), Coyer et al. (2022) and Pol‐Castañeda et al. (2022). Conversely, the appraisal also identified studies with lower scores, indicating areas for potential improvement in their methodological rigour. Most studies clustered in the mid‐to‐high range, suggesting generally adequate reporting and methodological quality across diverse study designs. However, most included studies were before‐and‐after studies, frequently conducted without control groups, which limits causal inference and hampers comparisons of effectiveness across studies. On the other hand, it is noticeable the difficulty in implementing pragmatic research into the real world of nurse‐led KT strategies.

### 3.6 Data Abstraction

A data extraction sheet was developed based on the Template for Intervention Description and Replication (TIDieR) (Hoffmann et al. 2014). The extracted data included: study title, lead author and publication year, country of study, study aim and design, theoretical rationale of the intervention, name of the nurse‐led intervention, providers, participants (target group), total number of participants, setting, materials and equipment used, procedures, delivery methods, intervention start and end dates, intervention and study duration, outcomes and measures, tailoring, modifications and results.

To ensure consistency in terminology, the term ‘pressure injury’ was used throughout the review, even when the original studies referred to ‘pressure ulcer’. This decision aligns with international taxonomies.

To ensure consistency and reliability in the data charting process, a pilot test was conducted using five studies selected to reflect variability in Knowledge Translation strategies and types of patient safety events. These studies were chosen to represent the main variations in patient safety events and Knowledge Translation strategies across different hospital settings. When studies lacked explicit statements about nursing leadership or Knowledge Translation strategy types, these were inferred from the authors' credentials, intervention descriptions or institutional roles.

### 3.7 Synthesis

The data were then transferred to Microsoft Excel to facilitate visualization, organization and categorization. NVivo, a qualitative analysis software, was used to support the narrative synthesis by organizing, coding and identifying patterns in the included studies.

To support consistency and transparency in categorizing types of knowledge translation strategies, the definitions of ‘educational’ and ‘multifaceted’ interventions were informed by existing taxonomies from the Expert Recommendations for Implementing Change (ERIC) framework (Proctor et al. 2013). In this review, strategies were classified as ‘educational’ when they focused primarily on a single‐component intervention aimed at improving knowledge or awareness (e.g., training sessions, workshops, educational materials). ‘Multifaceted’ strategies were defined as those that incorporated two or more components (e.g., education plus audit and feedback, simulation or use of digital tools). Although not all studies explicitly used these frameworks, their concepts were used as a reference for classification during data extraction and synthesis.

Data analysis employed an inductive approach, based on thematic analysis, to identify recurring patterns across the included studies. During the full‐text review, excerpts related to nurse‐led knowledge translation strategies were highlighted, particularly those concerning their characteristics, implementation methods, observed impacts and leadership components. Extracted units of meaning were systematically coded and grouped according to similarity. From this process, thematic categories were developed to represent the main findings, ensuring conceptual distinction among them and alignment with the review objectives. This analytical process enabled a descriptive synthesis structured into three thematic categories, presented in the results section.

## 4 Results

A total of 23,691 records were identified through database searches. The study selection process is illustrated in the PRISMA flow diagram (Figure 1). After applying the eligibility criteria, 59 studies met the eligibility requirements and were included in the review (File S4). These studies were conducted across 17 countries, primarily in the United States (*n* = 33), followed by Australia (*n* = 5), Brazil (*n* = 4), Spain (*n* = 3), Romania (*n* = 2) and Singapore (*n* = 2). One study was conducted in each of the following locations: Canada, China, Iceland, Indonesia, Portugal, Qatar, Sweden, Switzerland, Taiwan and Turkey.

**FIGURE 1:**
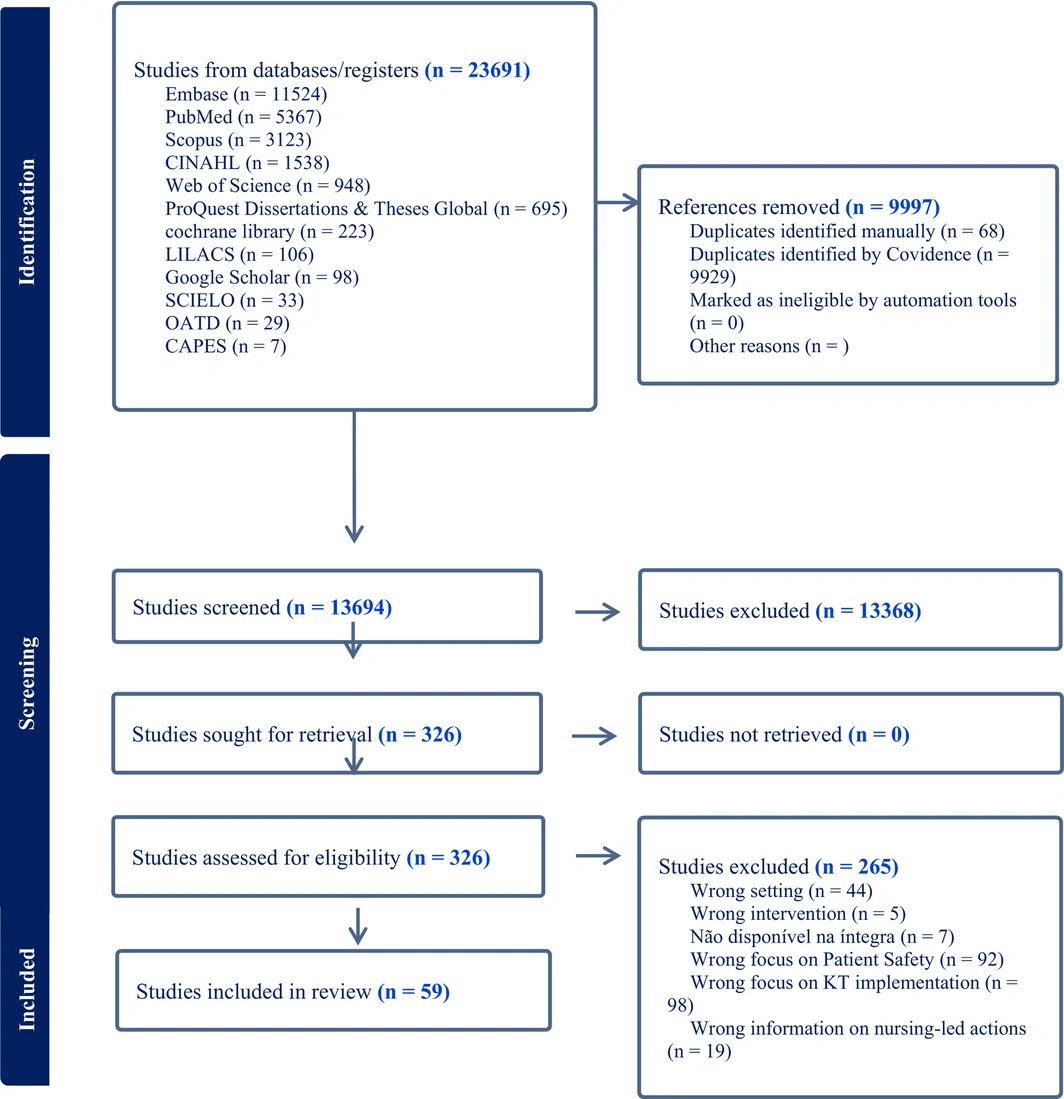
Flow chart of the study selection process.

The number of articles on Knowledge Translation strategies implemented for patient safety in hospitals has increased in recent years. A trend of rising publications over the years is observed, with a more pronounced growth starting in 2015. The period from 2018 to 2023 accounts for 61% of the publications (36 out of 59 articles). The average number of publications per year was 3.21 articles (59 publications over 19 years), with a median of three articles per year. The highest number of publications (*n* = 10) occurred in 2022. Most of the studies were before‐and‐after studies (*n* = 52), followed by cohort studies (*n* = 4) and randomized controlled trials (*n* = 3).

The Knowledge Translation (KT) strategies implemented to improve patient safety in hospitals primarily targeted adverse events, including: pressure injuries (*n* = 14), falls (*n* = 13), peripheral and central intravenous catheter‐related complications (*n* = 9), perioperative safety events (*n* = 4), hospital‐acquired pneumonia, including stroke‐associated pneumonia (*n* = 4), urinary catheter‐associated complications (*n* = 3), medication safety incidents (*n* = 1), management of aggression in patients with cognitive impairment (*n* = 1), and healthcare‐associated infections (HAIs) (*n* = 1).

Other strategies focused on safe practices and broader safety initiatives, such as hand hygiene adherence (*n* = 4), safety during care transitions (*n* = 3), initiatives to improve safety culture (*n* = 1), implementation of general best practices in healthcare (*n* = 1).

There was considerable heterogeneity in the duration of the interventions, ranging from 2 days (Ahsan et al. 2021) to 120 months (Mordiffi et al. 2016). Among the studies reporting this information (*n* = 33), the most frequently observed duration was 3 months (Nedelcu et al. 2022; Szymaniak 2015; Wright 2014). However, in 26 studies (Al Eid 2015; Akrour et al. 2022; Hoke and Guarracino 2016), the duration of the interventions was not specified, limiting comparisons across settings.

The extracted data included detailed information on the type of patient safety event targeted by each intervention, the nature of the knowledge translation strategy applied, the core components implemented, and the most relevant outcomes related to the review questions (File S3).

The data were organized into three thematic categories: (1) Characteristics and Implementation Processes of Nurse‐Led KT Strategies, (2) Impact of Knowledge Translation Strategies on Patient Safety and (3) Nursing Leadership Enabling KT.

### 4.1 Characteristics and Implementation Processes of Nurse‐Led KT Strategies

#### 4.1.1 Key Actors and Professional Roles

Fifteen studies highlighted the importance of specialized and experienced nurses in the development of Knowledge Translation strategies. For example, these professionals were referred to as Advanced Practice Nurses (APNs) (Pol‐Castañeda et al. 2022), Nurse Practitioners (NPs) (Liang et al. 2019), Clinical Nurse Specialists (CNSs) (Fabbruzzo‐Cota et al. 2016; Warren et al. 2019), Skin and Wound Specialists (Barakat‐Johnson et al. 2020; Brown‐Deveaux et al. 2022), Certified Nurse Researchers (Anderson et al. 2009) and Nurse Educators (Andreessen et al. 2012). Eleven studies also described the involvement of ‘champions’ acting as training facilitators (Dykes et al. 2019; Godlock et al. 2016; Wright 2014).

Knowledge Translation initiatives were implemented across diverse clinical environments, from long‐term care and intensive care units to surgical and emergency settings. This variety reflects the adaptability of KT approaches to different levels of clinical complexity, although many studies did not specify the exact setting, limiting contextual analysis.

Target populations varied widely, encompassing nursing staff, multidisciplinary teams, patients and family members. Most interventions focused on the nursing workforce, including care nurses, nursing technicians and specialist nurses, highlighting their central role in translating evidence into safer care (Ayala 2021; Bjorklund‐Lima et al. 2023; Szymaniak 2015). Other studies adopted a broader approach by promoting interprofessional collaboration that included physicians, physiotherapists, nutritionists, social workers and psychologists (Bartolowits et al. 2022; DiGerolamo and Chen‐Lim 2021; Roe and Williams 2014). Patient participation was also reported in diverse contexts and inpatient units (Coyer et al. 2022; Esaka 2020; Mansukhani et al. 2015) and some studies additionally engaged patients' families (de Maia et al. 2018; Choong et al. 2021; Mordiffi et al. 2016).

#### 4.1.2 Components and Tools of KT Strategies

It was observed that several studies incorporated simulation scenarios as part of their knowledge translation strategies across different areas of care. Specifically, the simulations included situational modules for catheter‐associated urinary tract infection (CAUTI) prevention (Liang et al. 2019); role‐playing exercises for standardized handoff communication using the IPASS framework (Brown‐Deveaux et al. 2022); testing of a sepsis screening tool using critical care scenarios (Roney et al. 2020); and other simulation‐based clinical interventions (Bjorklund‐Lima et al. 2023; Gupta et al. 2021; Godlock et al. 2016).

A total of 56 studies were identified as employing multifaceted strategies for Knowledge Translation. These strategies integrated various components, including education, training, simulation, care practices and audit with feedback mechanisms, to address complex healthcare challenges. In contrast, only three studies (Albornos‐Muñoz et al. 2018; Blondal et al. 2016; Warren et al. 2019) adopted one‐dimensional approaches, focusing exclusively on a single intervention type without incorporating additional complementary elements.

Technological resources played a complementary role in several KT interventions. Common tools included online educational modules (Coyer et al. 2022; Liu et al. 2022), email reminders with educational or reflective content (Abbott et al. 2006; Andreessen et al. 2012), and alert systems aimed at preventing adverse events such as patient falls (Bargmann and Brundrett 2020; McCarty et al. 2018) and pressure injuries (Avşar and Karadağ 2018). Real‐time monitoring and response technologies were also reported as part of KT strategies that facilitated the timely exchange and application of knowledge in clinical practice (Hoke and Guarracino 2016; Szymaniak 2015). Rather than serving as stand‐alone implementation mechanisms, these digital tools were integrated to strengthen the dissemination, accessibility and consistent application of evidence in nursing practice.

#### 4.1.3 Operational Processes and Stages of Implementation

Implementation processes were commonly supported by mechanisms that facilitated monitoring, adaptation and continuous improvement. In 27 studies, implementation components such as audit and feedback cycles, team discussions and workflow assessments were described (Ayala 2021; Erichsen Andersson et al. 2018; Fabbruzzo‐Cota et al. 2016). Team feedback sessions were used to identify practical challenges related to workload and workflow (Choong et al. 2021; Gupta et al. 2021; Liu et al. 2022), while regular audits monitored compliance with clinical guidelines and safety practices (Dykes et al. 2019; Albornos‐Muñoz et al. 2018; Esaka 2020).

In several cases, audit results were subsequently integrated into training and educational programs to reinforce evidence‐based practice and promote sustained behavioural change (Nedelcu et al. 2022; Coutinho et al. 2022; Rivera et al. 2020). Four studies implemented huddles and debriefs as reflective feedback strategies. For instance, huddles (Hoke and Guarracino 2016; Godlock et al. 2016) were used to analyse contributing factors, engage staff in real‐time discussions and propose evidence‐based solutions. Likewise, debriefs facilitated structured reflection, involving nurses, patients and their families.

Established implementation frameworks were often employed to guide KT processes. Among them, the Find–Organize–Clarify–Understand–Select (FOCUS) and Plan–Do–Check–Act (PDCA) cycles were applied to support iterative improvement (Godlock et al. 2016; Mordiffi et al. 2016). The Iowa Model of Evidence‐Based Practice (McCarty et al. 2018) and the Joanna Briggs Institute's Practical Application of Clinical Evidence System (PACES) were also frequently used (Nedelcu et al. 2022; Coutinho et al. 2022).

### 4.2 Impact of Knowledge Translation Strategies on Patient Safety

#### 4.2.1 Clinical Outcomes and Patient Safety Indicators

Most studies reported reductions in incidents, particularly falls. The observed decrease in falls ranged from 25% (Dykes et al. 2019) to 55%, with one study reporting a 72% reduction in falls with injuries (Hoke and Guarracino 2016). Regarding pressure injuries, reductions were reported from 3.4% to 0.48% (Rivera et al. 2020) and from 7.7% to 3.3% (Coyer et al. 2022). Additional outcomes included decreases in non‐ventilator‐associated hospital‐acquired pneumonia (NV‐HAP) (Liu et al. 2022), ventilator‐associated pneumonia (VAP) (Warren et al. 2019), medication errors (Al Eid 2015), peripheral intravenous catheter (PIVC) failures (Blanco‐Mavillard et al. 2021; Taylor 2015), catheter‐associated bloodstream infections (BSI) (Morgan and Thomas 2007) and sepsis‐related deaths (Roney et al. 2020).

Some studies did not identify significant impacts resulting from the interventions, including reductions in ventilator‐associated pneumonia (VAP) rates (Abbott et al. 2006), catheter‐associated urinary tract infections (Blondal et al. 2016) and adherence to recommended clinical practices, as well as no significant impact on the documentation of care plans (Albornos‐Muñoz et al. 2018). One study reported that the average rate of central line‐associated bloodstream infections (CLABSI) decreased by 22.8% following the intervention; however, one participating hospital recorded a 29% increase in these infections (Engel et al. 2023).

#### 4.2.2 Process Improvements and Adherence to Guidelines

Some studies, such as Akrour et al. (2022), reported improvements in the quality of procedures and the development of individualized care plans. Higher levels of compliance rates in central line maintenance were observed following the implementation of a compliance coach (Ayala 2021). Other studies also highlighted increased knowledge of and adherence to safe practices (Bjorklund‐Lima et al. 2023; Ahsan et al. 2021), as well as greater compliance with clinical guidelines, including the study by de Maia et al. (2018) among 10 others.

Evidence of improvements in the quality of care processes was reported in 22 studies, including workflow optimization through a reduction in steps, standardization of handover/SBAR and decreased response times (e.g., Albertini et al. 2023). Auditing and monitoring of bundles, using checklists and compliance panels, were described in 42 studies (e.g., Szymaniak 2015). Additionally, 32 studies reported enhanced compliance and adherence to clinical guidelines (e.g., Day 2009).

#### 4.2.3 Economic, Organizational and Cultural Impacts of KT Strategies

The safety culture was reported as being strengthened through engaged leadership and monthly audits (Hoke and Guarracino 2016; Godlock et al. 2016), as well as accountability and the use of transparent metrics to monitor outcomes (Andreessen et al. 2012; Barakat‐Johnson et al. 2020). Patient and family education played a crucial role in strengthening the safety culture (Dykes et al. 2019). These interventions were sustained by active leadership, continuous monitoring and multidisciplinary involvement.

Nurse‐led Knowledge Translation strategies reported positive economic impacts across different areas of patient safety. Notably, a potential cost saving of up to €19.50 per inserted PIVC was observed, associated with reduced device failure (Blanco‐Mavillard et al. 2021). In pressure injury prevention, annual cost savings of $837,387 (Barakat‐Johnson et al. 2020) and $379,767 (Gallo et al. 2020) were reported. Despite the high initial cost of preventive actions (Avşar and Karadağ 2018), there was a significant reduction in tissue deterioration and in the treatment costs related to pressure injuries. In the context of falls, video monitoring resulted in annual savings of $84,000 due to reduced use of sitters (Spano‐Szekely et al. 2019) and increasing the frequency of oral care in mechanically ventilated patients led to direct cost savings of over $21,000 (Eppling and Nickel 2023).

### 4.3 Nursing Leadership Enabling KT

The actions led by nurses were mainly focused on educational interventions and staff training, reported in 39 studies. For example, bedside education was provided by nurses specializing in skin integrity or by wound care specialists from the unit (Barakat‐Johnson et al. 2020). Nurses were also involved in the assessment and implementation of clinical protocols, described in 13 studies (Ballard et al. 2008). Several studies described nurses as responsible for conducting audits and monitoring clinical practice, reported in 19 studies (Dobre et al. 2023). Nurses also contributed to the development and delivery of educational content and support materials (Schumacher et al. 2013; Roe and Williams 2014), including the creation of Evidence‐Based Practice (EBP) modules and review of clinical procedures (Brown‐Deveaux et al. 2022).

In addition, nurses played key roles as project coordinators (de Maia et al. 2018), as advanced practice nurses or doctoral‐level nursing students (Mansukhani et al. 2015), and as facilitators in simulation‐based training sessions (de Almeida et al. 2022; Bartolowits et al. 2022; Godlock et al. 2016). In some studies, they were identified as members of interdisciplinary implementation teams (Dykes et al. 2019) or acted as compliance coaches (Ayala 2021) and knowledge brokers (de Maia et al. 2018). Educational leadership was also observed through nurse educators who supported knowledge uptake among healthcare teams (Spano‐Szekely et al. 2019).

## 5 Discussion

To our knowledge, this is among the first scoping reviews to examine nurse‐led Knowledge Translation strategies aimed at improving patient safety in hospital settings. The findings reveal an increase in publications since 2015, with most studies employing before‐and‐after designs across 17 countries. The reviewed interventions primarily targeted pressure injuries, patient falls and catheter‐related complications and were frequently associated with reductions in adverse events. Strategies combining multiple components such as education, simulation, audits and feedback were most common, with nurse protagonism in implementation. Key outcomes included improved adherence to clinical guidelines, workflow optimization and cost savings, though some studies reported variable or non‐significant effects.

Although nurse‐led knowledge translation (KT) strategies are often described as ‘multifaceted’, the analysed studies indicate that they cluster around a small number of strategic patterns with different underlying mechanisms. These include strategies focused on building clinical capacity (e.g., education and simulation), standardizing care processes (e.g., protocols, bundles and checklists), supporting ongoing feedback and learning (e.g., audits and debriefings), and those led through nursing leadership and governance. The widespread use of combined approaches appears to reflect a pragmatic response to the complexity of patient safety work in hospital settings.

Across the included studies, these patterns were associated with different types of outcomes. Capacity‐building strategies were mainly linked to improvements in knowledge, skills and short‐term adherence to safe practices, particularly in technically complex areas. Process‐standardization strategies were more often associated with reductions in nursing‐sensitive adverse events, such as falls and pressure injuries. Feedback‐based strategies were described as supporting behavioural change during the intervention period, while leadership‐mediated approaches were more frequently reported alongside organizational, cultural and economic outcomes. At the same time, each approach presented limitations, including reliance on skilled leadership, ongoing resource demands and challenges related to scalability, underscoring the importance of aligning strategy design with local context and long‐term support.

What distinguishes the nurse‐led KT strategies identified in this review is how nursing practice shapes the translation of evidence into everyday care. Nurses led these strategies through their continuous presence at the point of care, enabling the adaptation of evidence‐based interventions to workflow realities and sustained engagement with frontline teams. By positioning KT within core nursing work, the review indicates how leadership, decision‐making and frontline clinical judgement are mobilized. From an interpretive perspective, this framing helps explain how nurse‐led KT strategies have been associated with leadership roles, educational processes and governance structures within hospital settings.

The detailed analysis of the 59 included studies revealed an increase in the diversity and frequency of publications on the topic, particularly over the last 6 years. The geographical distribution of the studies revealed a predominance of research conducted in the United States (*n* = 33), followed by Australia (*n* = 5) and Brazil (*n* = 4). This concentration in high‐income countries reflects the current landscape of scientific production on the topic, with comparatively fewer studies from low‐ and middle‐income countries (LMICs) (Gomez et al. 2022). This finding is also in line with a study that identified Brazil as one of the six countries experiencing significant growth in scientific production, currently ranking among the top 20 in the world (Oliveira et al. 2022).

However, geographical imbalance can affect the applicability of KT strategies in different health contexts. This disparity highlights potential gaps in the volume of research conducted in developing nations. Studies suggest that Knowledge Translation contributes to bridging these gaps, helping to transform knowledge into practice, particularly for the underserved populations (World Health Organization 2005).

In resource‐limited hospital settings, structural constraints make it even more essential to align external evidence with local realities, ensuring that nurse‐led interventions are both feasible and sustainable. Simplifying the components of KT strategies and leveraging the existing capacities of nursing teams can optimize the use of scarce resources. Furthermore, prioritizing the generation of context‐specific evidence within hospitals helps identify locally relevant barriers and facilitators, making KT efforts more responsive to real needs and more effective in improving patient safety outcomes (Kalbarczyk et al. 2021; Parascandola et al. 2022).

The included studies employed a variety of strategy designs, with a predominance of multifaceted and context‐sensitive approaches. Simulations used in some studies (Godlock et al. 2016; Roney et al. 2020) were described as contributing to improvements in knowledge, skills and team behaviour outcomes and moderate effects on patient‐related outcomes (Cook et al. 2011), as well as a growing trend toward introducing new practices in the implementation of Knowledge Translation for patient safety (Gard et al. 2022).

Many studies explored multifaceted strategies, including the development of practice guidelines, audits, feedback and structured intervention plans, often integrated with consolidated models such as FOCUS‐PDCA (Godlock et al. 2016), JBI PACES (Albertini et al. 2023; Coutinho et al. 2022) and the Iowa Model (Bjorklund‐Lima et al. 2023; Dykes et al. 2019). These findings suggest a preference for evidence‐based approaches that are well founded, which underscores the need for strategies that extend beyond the mere dissemination of information, emphasizing the importance of interventions that foster the practical application of knowledge (LaRocca et al. 2012; Straus et al. 2009).

Technology was frequently integrated into knowledge translation strategies, mainly to support education, clinical monitoring, communication and risk prevention (Dykes et al. 2019; Fabbruzzo‐Cota et al. 2016; Roe and Williams 2014). Digital audits, alert systems and online educational platforms appeared to facilitate adherence to evidence‐based practices and sustained engagement. Although these tools may enhance care quality and reduce errors (Chaudhry et al. 2006; Harerimana and Mtshali 2020), their specific contribution to patient safety and the long‐term sustainability of interventions remains underexplored, warranting further investigation. Within the included studies, the use of digital tools can be interpreted as supporting KT processes by facilitating access to evidence, encouraging adherence to recommended practices and enabling feedback within clinical workflows.

Reflective feedback methods, both individual and collective, were reported in some studies as part of the implementation of knowledge translation strategies. These approaches included structured debriefings, team huddles and feedback sessions involving healthcare professionals, and in some cases, patients and families. The use of feedback was described as a mechanism to support ongoing engagement with clinical practices and to facilitate communication between team members, aligning with broader efforts to integrate evidence into routine care (Ivers et al. 2012).

Nurses' roles in developing and implementing strategies included coordinating action plans, facilitating educational sessions with staff, mediating communication between different professional groups, and overseeing adherence to evidence‐based protocols (Albornos‐Muñoz et al. 2018; Brown‐Deveaux et al. 2022; Godlock et al. 2016). In some studies, nurses led interdisciplinary efforts to adapt guidelines to local contexts (Barakat‐Johnson et al. 2020; DiGerolamo and Chen‐Lim 2021; Rivera et al. 2020), supported audit processes, and acted as local agents responsible for maintaining team engagement.

These professionals were often described not only as executors of planned activities but also as operational leaders who contributed to the continuity of interventions, contextual adaptation of practices and team mobilization throughout the process. These descriptions illustrate the importance of nursing leaders in facilitating and supporting the translation of knowledge into complex clinical contexts (Chan et al. 2023).

The identified adverse events are nursing‐sensitive indicators and serve as proxies for care quality, reflecting the overall quality of care. They underscore the role of nursing leadership in mitigating risks and promoting safe care practices (Cappelli et al. 2024) as they are in a key position to question what is done and utilize the most up‐to‐date research evidence in healthcare decision‐making (Cullen and Titler 2004).

Nurses were frequently described as central actors in the coordination and reception of Knowledge Translation strategies (Melnyk et al. 2018), a finding corroborated by the balanced number of studies focusing on nurses and patients. The predominance of studies including inpatients indicates prioritization of Knowledge Translation in direct care. However, only 19 studies included professionals from other health areas, showing a possible gap in the interprofessional approach. Effective communication and collaboration between professionals were essential for the dissemination (MacDonald et al. 2008), application and implementation of evidence‐based practices in the care environment.

The low frequency of studies aimed at family members indicates a gap in the implementation of Knowledge Translation strategies, despite the recognized contribution to effective continuity of care (Bombard et al. 2018). In addition, involving family members in interventions enhances communication between health professionals and caregivers, promoting a more collaborative and integrated approach to care, which can have a positive impact on clinical outcomes (Canadian Institutes of Health Research 2020; Grimshaw et al. 2012).

The review revealed that most studies reported a reduction in patient safety incidents following the implementation of nurse‐led knowledge translation strategies. In addition to these outcomes, several studies reported improvements in care processes, such as streamlined workflows, enhanced team communication and implementation of clinical protocols (Pol‐Castañeda et al. 2022; Roe and Williams 2014), as well as the development of individualized care plans (Akrour et al. 2022), lower hospital readmission rates (Mansukhani et al. 2015) and increased adherence to safe practices (Bjorklund‐Lima et al. 2023; Fabbruzzo‐Cota et al. 2016; Liang et al. 2019; Pol‐Castañeda et al. 2022).

However, some studies reported difficulties in maintaining these behavioural improvements over time, particularly in sustaining adherence to recommended practices (Abbott et al. 2006).

The sustainability of these interventions remains an open question, as there are few studies evaluating the long‐term maintenance of behavioural changes. This gap in literature reinforces the need for research investigating strategies to consolidate safe practices and ensure their continuity in the routine of health professionals (Descatha and Fadel 2023; Proctor et al. 2015).

This challenge reinforces the need to understand sustainability as an intrinsic component of the knowledge translation process, rather than merely a delayed outcome. Considering Nilsen's (2015) taxonomy, sustainability can be pursued more comprehensively by integrating multiple perspectives, including leadership engagement, the availability and management of resources and the use of implementation theories that explain how practices become consistently and durably embedded into the daily routines of healthcare teams.

Only one study addressed knowledge translation strategies involving implementation components targeting medication errors (Al Eid 2015). Given that such errors are among the leading causes of disability and mortality worldwide, their limited representation in the included studies points to a critical area for further investigation in future research.

### 5.1 Limitations

The findings of this scoping review should be interpreted with caution. The heterogeneity of study designs, reporting standards and contexts may also influence the interpretation of the findings. This limitation is particularly relevant for international readers seeking to translate this evidence into practice or policy.

Most evidence on nurse‐led Knowledge Translation strategies for patient safety originates from high‐income countries, with limited representation from low‐ and middle‐income countries. This geographical imbalance raises concerns about transferability, as systemic challenges, such as resource constraints, workforce shortages and weaker healthcare infrastructure, may limit direct applicability.

Future research should prioritize contextual adaptation and evaluate scalable, low‐cost interventions that leverage existing nursing capacity. Studies conducted in low‐ and middle‐income countries should be expanded to address the dynamics of local health systems, with particular attention to patient and family engagement throughout the implementation process, ensuring that interventions reflect community priorities and cultural realities. Long‐term sustainability needs to be assessed through ongoing follow‐up and monitoring, accompanied by comparative research on the effectiveness and cost‐effectiveness of different KT strategies across diverse practice settings. Furthermore, investigating the organizational impacts, such as institutional capacity and policy changes, and their influence on patient safety and quality of care, can help bridge the persistent gap between knowledge and practice, guiding equitable global improvements in healthcare delivery.

## 6 Conclusions

The Knowledge Translation strategies led by nurses were heterogeneous, often involving simulations, digital technologies and multifaceted approaches tailored to specific clinical contexts. These strategies reflect the complexity of bridging research and practice, encompassing not only the implementation of evidence‐based interventions but also the adaptation, dissemination and continuous evaluation of knowledge in hospital settings. Nurse leadership was consistently described as an enabling factor, supporting engagement, contextual alignment and the use of evidence‐informed practices.

The results suggest that nurse‐led KT strategies are frequently associated with reductions in adverse incidents and improvements in care processes, the development of individualized care plans and cost reductions. However, challenges remain in sustaining these interventions, particularly in maintaining behavioural changes, adherence to recommended practices and addressing the disproportionate concentration of studies in high‐income countries. This fact highlights significant gaps in research conducted in developing nations, pointing to the need for targeted efforts.

These findings highlight potential priorities for patient safety scholarship and practice, including strengthening nursing leadership capacity and evaluating strategies designed to support sustained use of evidence‐informed practices.

Taken together, these findings indicate that KT in patient safety should be understood not as a set of isolated strategies, but as a continuous, nurse‐led professional and organizational process. By foregrounding nursing leadership as a structural factor of nursing‐sensitive safety outcomes, this review advances current understanding of how evidence becomes embedded in hospital practice. Although the evidence is context‐dependent, the conclusions point to transferable and scalable principles, positioning investment in nursing leadership and KT capacity as a strategic pathway to improving quality, safety and efficiency in hospital settings internationally.

## Funding

This work was supported by Coordination for the Improvement of Higher Education Personnel (CAPES), Ministry of Education, Brazil [Finance Code 001].

## Ethics Statement

Ethical approval was not required for this scoping review as it involved the analysis of publicly available studies.

## Consent

The authors have nothing to report.

## Conflicts of Interest

The authors declare no conflicts of interest.

## Supporting information


**File S1:** jan70621‐sup‐0001‐FileS1.docx.

**File S2:** jan70621‐sup‐0002‐FileS2.xlsx.

**File S3:** jan70621‐sup‐0003‐FileS3.docx.

**File S4:** jan70621‐sup‐0004‐FileS4.docx.

## Data Availability

The data supporting the findings of this study are available from the corresponding author upon reasonable request.
