# Supplementary material for: Nursing‐Led Knowledge Translation Strategies to Improve Patient Safety in Hospital Settings: A Scoping Review
Source: J Adv Nurs. 2026 Apr 17;82(11):9595–608. doi: 10.1111/jan.70621 (PMC13661768; doi:10.1111/jan.70621)
Supplement: Supplementary file 1 [file JAN-82-9595-s001.docx]

*File S3*

**Table 3. Quality appraisal of the studies through the Quality Assessment of Diagnostic Accuracy Studies (QuADS) tool and scores.**

| **Authors and publication year** | **Final Score** |
| --- | --- |
| 1. Abbott et al. (2006) | 35 out of 39 |
| 2. Albornos-Munoz et al. (2018) | 36 out of 39 |
| 3. Adams (2022) | 37 out of 39 |
| 4. Ahsan et al. (2021) | 31 out of 29 |
| 5. Akinwole (2015) | 37 out of 39 |
| 6. Akrour et al. (2022) | 18 out of 30 |
| 7. Al Eid (2015) | 17 out of 30 |
| 8. Albertini et al. (2023) | 37 out of 39 |
| 9. Almeida et al. (2022) | 33 out of 39 |
| 10. Andersson et al. (2023) | 18 out of 30 |
| 11. Anderson et al. (2009) | 21 out of 39 |
| 12. Andreessen L. (2012) | 31 out of 39 |
| 13. Arcilla (2015) | 34 out of 39 |
| 14. Armour-Burton et al. (2013) | 18 out of 30 |
| 15. Avşar & Karadağ (2018) | 33 out of 39 |
| 16. Ayala (2019) | 35 out of 39 |
| 17. Ballard et al. (2007) | 14 out of 38 |
| 18. Barakat-Johnson et al. (2019) | 36 out of 39 |
| 19. Bargmann & Brundrett (2020) | 29 out of 39 |
| 20. Barker et al. (2012) | 38 out of 39 |
| 21. Bartolowits et al. (2023) | 28 out of 39 |
| 22. Bjorklund-Lima (2022) | 31 out of 39 |
| 23. Blanco-Mavillard (2023) | 39 out of 39 |
| 24. Blondal et al. (2016) | 38 out of 39 |
| 25. Brown-Deveaux et al. (2022) | 24 out of 39 |
| 26. Choong et al. (2022) | 39 out of 39 |
| 27. Coutinho et al. (2022) | 36 out of 39 |
| 28. Coyer et al. (2021) | 39 out of 39 |
| 29. Day C. (2009) | 17 out of 39 |
| 30. DiGerolamo et al. (2021) | 33 out of 39 |
| 31. Dobre et al. (2022) | 38 out o 39 |
| 32. Dykes et al. (2019) | 9 out of 21 |
| 33. Engel et al. (2023) | 37 out of 39 |
| 34. Eppling & Nickel (2023) | 28 out of 39 |
| 35. Esaka (2020) | 38 out of 39 |
| 36. Fabbruzzo-Cota et al. (2016) | 34 out of 39 |
| 37. Gallo et al. (2019) | 32 out of 39 |
| 38. Godlock et al. (2016) | 32 out of 39 |
| 39. Gupta et al. (2021) | 38 out of 39 |
| 40. Hoke & Guarracino (2016) | 26 out of 39 |
| 41. Liang et al. (2019) | 36 out of 39 |
| 42. Liu et al. (2022) | 37 out of 39 |
| 43. Lopez et al. (2004) | 33 out of 39 |
| 44. Maia et al. (2018) | 35 out of 39 |
| 45. McCarty et al. (2018) | 25 out of 39 |
| 46. Melnyk et al. (2017) | 30 out of 39 |
| 47. Mordiffi et al. (2016) | 30 out of 39 |
| 48. Morgan & Thomas (2007) | 25 out of 39 |
| 49. Nedelcu et al. (2022) | 38 out of 39 |
| 50. Pol-Castañeda et al. (2022) | 39 out of 39 |
| 51. Riveira et al. (2020) | 28 out of 39 |
| 52. Roe & Williams (2014) | 22 out of 39 |
| 53. Roney et al. (2019) | 33 out of 39 |
| 54. Schumacher et al. (2013) | 22 out of 39 |
| 55. Spano-Szekely et al. (2019) | 31 out of 38 |
| 56. Szymaniak (2013) | 31 out of 38 |
| 57. Taylor (2015) | 31 out of 38 |
| 58. Warren et al. (2019) | 34 out of 39 |
| 59. Wright et al. (2014) | 35 out of 39 |
