# Supplementary material for: Nursing‐Led Knowledge Translation Strategies to Improve Patient Safety in Hospital Settings: A Scoping Review
Source: J Adv Nurs. 2026 Apr 17;82(11):9595–608. doi: 10.1111/jan.70621 (PMC13661768; doi:10.1111/jan.70621)
Supplement: Supplementary file 2 [file JAN-82-9595-s002.docx]

*File S1*

***PubMed/MEDLINE***

***("Translational Science, Biomedical"[Mesh]*** *OR "Biomedical Translational Science" OR "Biomedical Translational Sciences" OR "Translational Medical Science" OR "Translational Medical Sciences" OR "Translational Medicine" OR "Translational Science" OR "Translational Sciences" OR "Biomedical Translation" OR "Biomedical Translations" OR "Knowledge Translation" OR "Knowledge Translations" OR "Knowledge transfer" OR "Knowledge brokering" OR "Knowledge management" OR "evidence-based practice interventions" OR "Knowledge sharing" OR "Knowledge Integration" OR "Translational Research" OR "Evidence-Based Practice" OR "Knowledge Application" OR "Research Utilization" OR "Knowledge Exchange" OR "Knowledge Dissemination" OR "Knowledge Mobilization") AND* ***("Patient Safety"[Mesh]*** *OR "Patient Safety" OR "Patients Safety" OR "Safety" OR "Safeties" OR "Patient" OR "Safety Management" OR "Safety culture" OR "Culture of safety" OR "Safety climate" OR "Quality" OR "Care quality" OR "Quality of care" OR "Care quality outcomes" OR "Patient Harm" OR "Patient outcome" OR "Adverse event" OR "Harmful incident" OR "Healthcare-associated harm" OR "Hazard" OR "Reportable circumstance" OR "Near miss" OR "No harm incident" OR "Adverse reaction" OR "Side effect" OR "Preventable" OR "Patient safety incident" OR "Harm" OR "Injury" OR "Disability" OR "Degree of harm" OR "Violation" OR "Risk" OR "Actions taken to reduce risk" OR "Never event") AND* ***("Hospital Units"[Mesh]*** *OR* ***"Hospitals"[Mesh]*** *OR* ***"Hospitals"*** *OR* ***"****Hospital****")*** *AND* ***("Nursing"[Mesh]*** *OR* ***"Nursing"*** *OR Nurs* OR* ***"Nurses"[Mesh]*** *OR* ***"Nurses")***

*Number of results: 5342*

***Embase (Elsevier)***

*("Biomedical Translational Science" OR "Biomedical Translational Sciences" OR "Translational Medical Science" OR "Translational Medical Sciences" OR "Translational Medicine" OR "Translational Science" OR "Translational Sciences" OR "Biomedical Translation" OR "Biomedical Translations" OR "Knowledge Translation" OR "Knowledge Translations" OR "Knowledge transfer" OR "Knowledge brokering" OR "Knowledge management" OR "evidence-based practice interventions" OR "Knowledge sharing" OR "Knowledge Integration" OR "Translational Research" OR "Evidence-Based Practice" OR "Knowledge Application" OR "Research Utilization" OR "Knowledge Exchange" OR "Knowledge Dissemination" OR "Knowledge Mobilization") AND* ***("Patient Safety"*** *OR "Patients Safety" OR "Safety" OR "Safeties" OR "Patient" OR "Safety Management" OR "Safety culture" OR "Culture of safety" OR "Safety climate" OR "Quality" OR "Care quality" OR "Quality of care" OR "Care quality outcomes" OR "Patient Harm" OR "Patient outcome" OR "Adverse event" OR "Harmful incident" OR "Healthcare-associated harm" OR "Hazard" OR "Reportable circumstance" OR "Near miss" OR "No harm incident" OR "Adverse reaction" OR "Side effect" OR "Preventable" OR "Patient safety incident" OR "Harm" OR "Injury" OR "Disability" OR "Degree of harm" OR "Violation" OR "Risk" OR "Actions taken to reduce risk" OR "Never event") AND* ***("Hospitals"*** *OR* ***"****Hospital****")*** *AND* ***("Nursing"*** *OR Nurs* OR* ***"Nurses")***

*all fields (lápis)*

*Number of results: 10.429*

***CINAHL (EBSCO)***

*("Biomedical Translational Science" OR "Biomedical Translational Sciences" OR "Translational Medical Science" OR "Translational Medical Sciences" OR "Translational Medicine" OR "Translational Science" OR "Translational Sciences" OR "Biomedical Translation" OR "Biomedical Translations" OR "Knowledge Translation" OR "Knowledge Translations" OR "Knowledge transfer" OR "Knowledge brokering" OR "Knowledge management" OR "evidence-based practice interventions" OR "Knowledge sharing" OR "Knowledge Integration" OR "Translational Research" OR "Evidence-Based Practice" OR "Knowledge Application" OR "Research Utilization" OR "Knowledge Exchange" OR "Knowledge Dissemination" OR "Knowledge Mobilization") AND* ***("Patient Safety"*** *OR "Patients Safety" OR "Safety" OR "Safeties" OR "Patient" OR "Safety Management" OR "Safety culture" OR "Culture of safety" OR "Safety climate" OR "Quality" OR "Care quality" OR "Quality of care" OR "Care quality outcomes" OR "Patient Harm" OR "Patient outcome" OR "Adverse event" OR "Harmful incident" OR "Healthcare-associated harm" OR "Hazard" OR "Reportable circumstance" OR "Near miss" OR "No harm incident" OR "Adverse reaction" OR "Side effect" OR "Preventable" OR "Patient safety incident" OR "Harm" OR "Injury" OR "Disability" OR "Degree of harm" OR "Violation" OR "Risk" OR "Actions taken to reduce risk" OR "Never event") AND* ***("Hospitals"*** *OR* ***"****Hospital****")*** *AND* ***("Nursing"*** *OR Nurs* OR* ***"Nurses")***

*Number of results: 1.549*

***Cochrane Library***

*("Biomedical Translational Science" OR "Biomedical Translational Sciences" OR "Translational Medical Science" OR "Translational Medical Sciences" OR "Translational Medicine" OR "Translational Science" OR "Translational Sciences" OR "Biomedical Translation" OR "Biomedical Translations" OR "Knowledge Translation" OR "Knowledge Translations" OR "Knowledge transfer" OR "Knowledge brokering" OR "Knowledge management" OR "evidence-based practice interventions" OR "Knowledge sharing" OR "Knowledge Integration" OR "Translational Research" OR "Evidence-Based Practice" OR "Knowledge Application" OR "Research Utilization" OR "Knowledge Exchange" OR "Knowledge Dissemination" OR "Knowledge Mobilization") AND* ***("Patient Safety"*** *OR "Patients Safety" OR "Safety" OR "Safeties" OR "Patient" OR "Safety Management" OR "Safety culture" OR "Culture of safety" OR "Safety climate" OR "Quality" OR "Care quality" OR "Quality of care" OR "Care quality outcomes" OR "Patient Harm" OR "Patient outcome" OR "Adverse event" OR "Harmful incident" OR "Healthcare-associated harm" OR "Hazard" OR "Reportable circumstance" OR "Near miss" OR "No harm incident" OR "Adverse reaction" OR "Side effect" OR "Preventable" OR "Patient safety incident" OR "Harm" OR "Injury" OR "Disability" OR "Degree of harm" OR "Violation" OR "Risk" OR "Actions taken to reduce risk" OR "Never event") AND* ***("Hospitals"*** *OR* ***"****Hospital****")*** *AND* ***("Nursing"*** *OR Nurs* OR* ***"Nurses")***

*Number of results: 3 / 218 trials*

***Scopus (Elsevier)***

*("Biomedical Translational Science" OR "Biomedical Translational Sciences" OR "Translational Medical Science" OR "Translational Medical Sciences" OR "Translational Medicine" OR "Translational Science" OR "Translational Sciences" OR "Biomedical Translation" OR "Biomedical Translations" OR "Knowledge Translation" OR "Knowledge Translations" OR "Knowledge transfer" OR "Knowledge brokering" OR "Knowledge management" OR "evidence-based practice interventions" OR "Knowledge sharing" OR "Knowledge Integration" OR "Translational Research" OR "Evidence-Based Practice" OR "Knowledge Application" OR "Research Utilization" OR "Knowledge Exchange" OR "Knowledge Dissemination" OR "Knowledge Mobilization") AND* ***("Patient Safety"*** *OR "Patients Safety" OR "Safety" OR "Safeties" OR "Patient" OR "Safety Management" OR "Safety culture" OR "Culture of safety" OR "Safety climate" OR "Quality" OR "Care quality" OR "Quality of care" OR "Care quality outcomes" OR "Patient Harm" OR "Patient outcome" OR "Adverse event" OR "Harmful incident" OR "Healthcare-associated harm" OR "Hazard" OR "Reportable circumstance" OR "Near miss" OR "No harm incident" OR "Adverse reaction" OR "Side effect" OR "Preventable" OR "Patient safety incident" OR "Harm" OR "Injury" OR "Disability" OR "Degree of harm" OR "Violation" OR "Risk" OR "Actions taken to reduce risk" OR "Never event") AND* ***("Hospitals"*** *OR* ***"****Hospital****")*** *AND* ***("Nursing"*** *OR Nurs* OR* ***"Nurses")***

*Number of results: 3.116*

***Web of Science (Clarivate Analytics)***

*("Biomedical Translational Science" OR "Biomedical Translational Sciences" OR "Translational Medical Science" OR "Translational Medical Sciences" OR "Translational Medicine" OR "Translational Science" OR "Translational Sciences" OR "Biomedical Translation" OR "Biomedical Translations" OR "Knowledge Translation" OR "Knowledge Translations" OR "Knowledge transfer" OR "Knowledge brokering" OR "Knowledge management" OR "evidence-based practice interventions" OR "Knowledge sharing" OR "Knowledge Integration" OR "Translational Research" OR "Evidence-Based Practice" OR "Knowledge Application" OR "Research Utilization" OR "Knowledge Exchange" OR "Knowledge Dissemination" OR "Knowledge Mobilization") AND* ***("Patient Safety"*** *OR "Patients Safety" OR "Safety" OR "Safeties" OR "Patient" OR "Safety Management" OR "Safety culture" OR "Culture of safety" OR "Safety climate" OR "Quality" OR "Care quality" OR "Quality of care" OR "Care quality outcomes" OR "Patient Harm" OR "Patient outcome" OR "Adverse event" OR "Harmful incident" OR "Healthcare-associated harm" OR "Hazard" OR "Reportable circumstance" OR "Near miss" OR "No harm incident" OR "Adverse reaction" OR "Side effect" OR "Preventable" OR "Patient safety incident" OR "Harm" OR "Injury" OR "Disability" OR "Degree of harm" OR "Violation" OR "Risk" OR "Actions taken to reduce risk" OR "Never event") AND* ***("Hospitals"*** *OR* ***"****Hospital****")*** *AND* ***("Nursing"*** *OR Nurs* OR* ***"Nurses")***

*Number of results: 945*

***LILACS / BDENF***

*("Biomedical Translational Science" OR "Biomedical Translational Sciences" OR "Translational Medical Science" OR "Translational Medical Sciences" OR "Translational Medicine" OR "Translational Science" OR "Translational Sciences" OR "Biomedical Translation" OR "Biomedical Translations" OR "Knowledge Translation" OR "Knowledge Translations" OR "Knowledge transfer" OR "Knowledge brokering" OR "Knowledge management" OR "evidence-based practice interventions" OR "Knowledge sharing" OR "Knowledge Integration" OR "Translational Research" OR "Evidence-Based Practice" OR "Knowledge Application" OR "Research Utilization" OR "Knowledge Exchange" OR "Knowledge Dissemination" OR "Knowledge Mobilization" OR "Ciência Translacional Biomédica" OR "Ciência Médica Translacional" OR "Ciência Translacional" OR "Medicina Translacional" OR "Tradução Biomédica" OR "Tradução de Conhecimento" OR "Tradução do Conhecimento" OR "Translação Biomédica" OR "Translação de Conhecimento" OR "Translação do Conhecimento" OR "Transferência de conhecimento" OR "Intermediação de conhecimento" OR "Gestão do conhecimento" OR "intervenções práticas baseadas em evidências" OR "Compartilhamento de conhecimento" OR "Integração de Conhecimento" OR "Pesquisa Translacional" OR "Prática Baseada em Evidências" OR "Aplicação de conhecimento" OR "Troca de conhecimento" OR "Disseminação de Conhecimento" OR "Mobilização de Conhecimento" OR "Ciencia Traslacional Biomédica" OR "ciencia médica traslacional" OR "investigación traslacional" OR "medicina traslacional" OR "traslación biomédica" OR "traslación de conocimientos" OR "Transferencia de conocimiento" OR "Intermediación del conocimiento" OR "Intervenciones prácticas basadas en evidencia" OR "Intercambio de conocimientos" OR "Integración de conocimientos" OR "Investigación traslacional" OR "Practica basada en la evidencia" OR "Aplicación del conocimiento" OR "Intercambio de conocimiento" OR "Difusión del conocimiento" OR "Movilización del conocimiento") AND* ***("Patient Safety"*** *OR "Patients Safety" OR "Safety" OR "Safeties" OR "Patient" OR "Safety Management" OR "Safety culture" OR "Culture of safety" OR "Safety climate" OR "Quality" OR "Care quality" OR "Quality of care" OR "Care quality outcomes" OR "Patient Harm" OR "Patient outcome" OR "Adverse event" OR "Harmful incident" OR "Healthcare-associated harm" OR "Hazard" OR "Reportable circumstance" OR "Near miss" OR "No harm incident" OR "Adverse reaction" OR "Side effect" OR "Preventable" OR "Patient safety incident" OR "Harm" OR "Injury" OR "Disability" OR "Degree of harm" OR "Violation" OR "Risk" OR "Actions taken to reduce risk" OR "Never event" OR "Segurança do Paciente" OR "Segurança" OR "Paciente" OR "Qualidade" OR "Evento adverso" OR "Incidente prejudicial" OR "Danos associados aos cuidados de saúde" OR "Notificação" OR "Quase acidente" OR "Nenhum incidente prejudicial" OR "Reação adversa" OR "Efeito colateral" OR "Evitável" OR "Erro" OR "Lesão" OR "Incapacidade" OR "Grau de dano" OR "Violação" OR "Risco" OR "Seguridad del Paciente" OR "Seguridad" OR "Calidad" OR "Daño al paciente" OR "Acontecimiento adverso" OR "incidente dañino" OR "Daños asociados con los cuidados de la salud" OR "Notificación" OR "Casi accidente" OR "Sin incidentes perjudiciales" OR "Reacción adversa" OR "Efecto colateral" OR "Evitable" OR "Error" OR "Lesión" OR "Incapacidad" OR "Grado de daño" OR "Violación" OR "Riesgo") AND* ***("Hospitals"*** *OR* ***"****Hospital****"*** *OR* ***"Unidades Hospitalares"*** *OR* ***"Hospitais"*** *OR* ***"****Hospitalar****"*** *OR* ***"Unidades Hospitalarias"*** *OR* ***"Hospitales")*** *AND* ***("Nursing"*** *OR Nurs* OR* ***"Nurses"*** *OR* ***"Enfermagem"*** *OR enfermeir* OR* ***"Enfermeria"*** *OR enfermer*)*

*Number of results LILACS: 85*

*Number of results BDENF: 84*

***SciELO***

*("Biomedical Translational Science" OR "Biomedical Translational Sciences" OR "Translational Medical Science" OR "Translational Medical Sciences" OR "Translational Medicine" OR "Translational Science" OR "Translational Sciences" OR "Biomedical Translation" OR "Biomedical Translations" OR "Knowledge Translation" OR "Knowledge Translations" OR "Knowledge transfer" OR "Knowledge brokering" OR "Knowledge management" OR "evidence-based practice interventions" OR "Knowledge sharing" OR "Knowledge Integration" OR "Translational Research" OR "Evidence-Based Practice" OR "Knowledge Application" OR "Research Utilization" OR "Knowledge Exchange" OR "Knowledge Dissemination" OR "Knowledge Mobilization" OR "Ciência Translacional Biomédica" OR "Ciência Médica Translacional" OR "Ciência Translacional" OR "Medicina Translacional" OR "Tradução Biomédica" OR "Tradução de Conhecimento" OR "Tradução do Conhecimento" OR "Translação Biomédica" OR "Translação de Conhecimento" OR "Translação do Conhecimento" OR "Transferência de conhecimento" OR "Intermediação de conhecimento" OR "Gestão do conhecimento" OR "intervenções práticas baseadas em evidências" OR "Compartilhamento de conhecimento" OR "Integração de Conhecimento" OR "Pesquisa Translacional" OR "Prática Baseada em Evidências" OR "Aplicação de conhecimento" OR "Troca de conhecimento" OR "Disseminação de Conhecimento" OR "Mobilização de Conhecimento" OR "Ciencia Traslacional Biomédica" OR "ciencia médica traslacional" OR "investigación traslacional" OR "medicina traslacional" OR "traslación biomédica" OR "traslación de conocimientos" OR "Transferencia de conocimiento" OR "Intermediación del conocimiento" OR "Intervenciones prácticas basadas en evidencia" OR "Intercambio de conocimientos" OR "Integración de conocimientos" OR "Investigación traslacional" OR "Practica basada en la evidencia" OR "Aplicación del conocimiento" OR "Intercambio de conocimiento" OR "Difusión del conocimiento" OR "Movilización del conocimiento") AND* ***("Patient Safety"*** *OR "Patients Safety" OR "Safety" OR "Safeties" OR "Patient" OR "Safety Management" OR "Safety culture" OR "Culture of safety" OR "Safety climate" OR "Quality" OR "Care quality" OR "Quality of care" OR "Care quality outcomes" OR "Patient Harm" OR "Patient outcome" OR "Adverse event" OR "Harmful incident" OR "Healthcare-associated harm" OR "Hazard" OR "Reportable circumstance" OR "Near miss" OR "No harm incident" OR "Adverse reaction" OR "Side effect" OR "Preventable" OR "Patient safety incident" OR "Harm" OR "Injury" OR "Disability" OR "Degree of harm" OR "Violation" OR "Risk" OR "Actions taken to reduce risk" OR "Never event" OR "Segurança do Paciente" OR "Segurança" OR "Paciente" OR "Qualidade" OR "Evento adverso" OR "Incidente prejudicial" OR "Danos associados aos cuidados de saúde" OR "Notificação" OR "Quase acidente" OR "Nenhum incidente prejudicial" OR "Reação adversa" OR "Efeito colateral" OR "Evitável" OR "Erro" OR "Lesão" OR "Incapacidade" OR "Grau de dano" OR "Violação" OR "Risco" OR "Seguridad del Paciente" OR "Seguridad" OR "Calidad" OR "Daño al paciente" OR "Acontecimiento adverso" OR "incidente dañino" OR "Daños asociados con los cuidados de la salud" OR "Notificación" OR "Casi accidente" OR "Sin incidentes perjudiciales" OR "Reacción adversa" OR "Efecto colateral" OR "Evitable" OR "Error" OR "Lesión" OR "Incapacidad" OR "Grado de daño" OR "Violación" OR "Riesgo") AND* ***("Hospitals"*** *OR* ***"****Hospital****"*** *OR* ***"Unidades Hospitalares"*** *OR* ***"Hospitais"*** *OR* ***"****Hospitalar****"*** *OR* ***"Unidades Hospitalarias"*** *OR* ***"Hospitales")*** *AND* ***("Nursing"*** *OR Nurs* OR* ***"Nurses"*** *OR* ***"Enfermagem"*** *OR enfermeir* OR* ***"Enfermeria"*** *OR enfermer*)*

*Number of results: 33*

***ProQuest Dissertations & Theses Global (PQDT Global)***

*noft(("Biomedical Translational Science" OR "Biomedical Translational Sciences" OR "Translational Medical Science" OR "Translational Medical Sciences" OR "Translational Medicine" OR "Translational Science" OR "Translational Sciences" OR "Biomedical Translation" OR "Biomedical Translations" OR "Knowledge Translation" OR "Knowledge Translations" OR "Knowledge transfer" OR "Knowledge brokering" OR "Knowledge management" OR "evidence-based practice interventions" OR "Knowledge sharing" OR "Knowledge Integration" OR "Translational Research" OR "Evidence-Based Practice" OR "Knowledge Application" OR "Research Utilization" OR "Knowledge Exchange" OR "Knowledge Dissemination" OR "Knowledge Mobilization") AND ("Patient Safety" OR "Patients Safety" OR "Safety" OR "Safeties" OR "Patient" OR "Safety Management" OR "Safety culture" OR "Culture of safety" OR "Safety climate" OR "Quality" OR "Care quality" OR "Quality of care" OR "Care quality outcomes" OR "Patient Harm" OR "Patient outcome" OR "Adverse event" OR "Harmful incident" OR "Healthcare-associated harm" OR "Hazard" OR "Reportable circumstance" OR "Near miss" OR "No harm incident" OR "Adverse reaction" OR "Side effect" OR "Preventable" OR "Patient safety incident" OR "Harm" OR "Injury" OR "Disability" OR "Degree of harm" OR "Violation" OR "Risk" OR "Actions taken to reduce risk" OR "Never event") AND ("Hospitals" OR "Hospital") AND ("Nursing" OR Nurs* OR "Nurses"))*

*Number of results: 395*

***Open Access Theses and Dissertations (OATD)***

*("Biomedical Translational Science" OR "Biomedical Translational Sciences" OR "Translational Medical Science" OR "Translational Medical Sciences" OR "Translational Medicine" OR "Translational Science" OR "Translational Sciences" OR "Biomedical Translation" OR "Biomedical Translations" OR "Knowledge Translation" OR "Knowledge Translations" OR "Knowledge transfer" OR "Knowledge brokering" OR "Knowledge management" OR "evidence-based practice interventions" OR "Knowledge sharing" OR "Knowledge Integration" OR "Translational Research" OR "Evidence-Based Practice" OR "Knowledge Application" OR "Research Utilization" OR "Knowledge Exchange" OR "Knowledge Dissemination" OR "Knowledge Mobilization") AND* ***("Patient Safety"*** *OR "Patients Safety" OR "Safety" OR "Safeties" OR "Patient" OR "Safety Management" OR "Safety culture" OR "Culture of safety" OR "Safety climate" OR "Quality" OR "Care quality" OR "Quality of care" OR "Care quality outcomes" OR "Patient Harm" OR "Patient outcome" OR "Adverse event" OR "Harmful incident" OR "Healthcare-associated harm" OR "Hazard" OR "Reportable circumstance" OR "Near miss" OR "No harm incident" OR "Adverse reaction" OR "Side effect" OR "Preventable" OR "Patient safety incident" OR "Harm" OR "Injury" OR "Disability" OR "Degree of harm" OR "Violation" OR "Risk" OR "Actions taken to reduce risk" OR "Never event") AND* ***("Hospitals"*** *OR* ***"****Hospital****")*** *AND* ***("Nursing"*** *OR Nurs* OR* ***"Nurses")***

*Number of results: 28*

***Catálogo de Teses e Dissertações (CAPES)***

*Tradução de Conhecimento AND Segurança do Paciente*

*Number of results: 15*

***Google Scholar***

*("Knowledge Translation" OR "Tradução do Conhecimento" OR "traslación de conocimientos") AND* ***("Patient Safety"*** *OR "Segurança do Paciente" OR "Seguridad del Paciente") AND* ***("Hospitals"*** *OR* ***"Hospitais")*** *AND* ***("Nursing"*** *OR* ***"Enfermagem"*** *OR* ***"Enfermeria")***

*Number of results: 4.280*

*For analysis purposes, only the first 10 pages were exported.*
