# Supplementary material for: Nursing‐Led Knowledge Translation Strategies to Improve Patient Safety in Hospital Settings: A Scoping Review
Source: J Adv Nurs. 2026 Apr 17;82(11):9595–608. doi: 10.1111/jan.70621 (PMC13661768; doi:10.1111/jan.70621)
Supplement: Supplementary file 3 [file JAN-82-9595-s004.docx]

*File S4*

***Table 2.*** *Characteristics, strategies, and outcomes of nurse-led knowledge translation interventions to improve patient safety in hospital settings.*

| ***Authors and publication year*** | ***Country of study*** | ***Type of safety event targeted*** | ***Intervention complexity*** | ***Intervention components*** | ***Outcomes*** |
| --- | --- | --- | --- | --- | --- |
| ***Abbott et al. (2006)*** | *United States* | *hospital-acquired pneumonia* | *Multifaceted* | *Development of clinical guidelines, educational packages, bedside reminders, and regular audits.* | *Occasional reduction in VAP rates in some ICUs; challenges in sustained adoption of guidelines.* |
| ***Adams (2022)*** | *United States* | *Perioperative safety* | *Multifaceted* | *Implementation of the "Sign Out" phase using checklists to reduce intraoperative specimen errors; surgical team training.* | *Specimen error rate reduced from 0.6% to 0.2%; improved communication and interprofessional collaboration.* |
| ***Ahsan et al. (2021)*** | *Indonesia* | *Healthcare-Associated Infections (HAI)* | *Educational* | *Knowledge management-based training, adherence to six steps and five moments of hand hygiene. Included pre- and post-tests to assess knowledge.* | *38% increase in nurses' knowledge and improved hand hygiene adherence.* |
| ***Akinwole (2015)*** | *USA* | *Peripheral and central intravenous catheter-related complications* | *Multifaceted* | *Policy updates, educational bundles, regular audits.* | *CLABSI rate dropped from 5.2 to 0 per 1,000 catheter days.* |
| ***Akrour et al. (2022)*** | *Switzerland* | *Aggression management in patients with cognitive impairment* | *Multifaceted* | *Training, implementation of the CMAI tool to identify at-risk patients, interdisciplinary case discussions in meetings.* | *Improved identification of triggers for aggression/agitation (7% → 46%).* |
| ***Al Eid (2015)*** | *United States* | *Medication Safety* | *Multifaceted* | *Medication safety committee, evidence-based policy updates, bedside rounds including patients in infusion checks.* | *Significant reduction in harmful medication errors. Ongoing reporting of near misses (e.g., one error and seven near misses in one month).* |
| ***Albertini et al. (2023)*** | *Brazil* | *Patient falls* | *Multifaceted* | *Online training, institutional protocol review, and customization in electronic records.* | *>75% compliance with key criteria; training reached 100% of staff.* |
| ***Albornos-Munoz et al. (2018)*** | *Spain* | *Patient falls* | *Educational* | *Fall prevention training, audits using JBI PACES, continuous feedback.* | *Compliance increased to 33.9%; non-injury falls remained during follow-up.* |
| ***Almeida et al. (2022)*** | *Brazil* | *Peripheral and central intravenous catheter-related complications* | *Multifaceted* | *Lecture and simulation on flushing and push-pause techniques.* | *Obstruction reduced from 20.4% to 4.5% (p<0.001); infiltration increased from 9.4% to 17.8% (p=0.023).* |
| ***Andersson et al. (2023)*** | *Sweden* | *Hand Hygiene* | *Iterative* | *Iterative co-creation of standard operating procedures (SOPs), monitoring hand hygiene guideline adherence, and aseptic technique.* | *Increased awareness of healthcare-associated infections (HAIs) and safety practices in the surgical setting.* |
| ***Anderson et al. (2009)*** | *Sweden* | *Best Practices in Healthcare* | *Multifaceted* | *Facilitated interprofessional dialogue, SOP co-creation, visualization of postoperative complication data, and “Learning Lab” sessions.* | *Increased awareness of organizational culture and individual practices; positive impact on HAI and HH dialogue.* |
| ***Andreessen L. (2012)*** | *United States* | *Urinary catheter-associated complications* | *Multifaceted* | *Education on preventive bundles, electronic documentation training, templates for insertion/removal, continuous feedback.* | *71% reduction in catheter days; complete documentation increased to 98%.* |
| ***Arcilla (2015)*** | *United States* | *Safety during care transitions* | *Multifaceted* | *Structured education in workshops and seminars for nurses on preventing surgical wound infection-related readmissions.* | *Readmission rate dropped from 14.3% to 0% when comparing the same periods in 2014 and 2015.* |
| ***Armour-Burton et al. (2013)*** | *USA* | *Pressure Injury* | *Multifaceted* | *Self-instructional educational modules, bimonthly audits.* | *Zero prevalence in 17 of the 20 quarters evaluated after implementation of the Healthy Skin Project.* |
| ***Avşar & Karadağ (2018)*** | *Turkey* | *Pressure Injury* | *Multifaceted* | *Evidence-based training to improve tissue tolerance; gradual implementation of preventive interventions.* | *Overall PI incidence reduction: intervention group = 13%, control = 33%.* |
| ***Ayala (2019)*** | *USA* | *Peripheral and central intravenous catheter-related complications* | *Multifaceted* | *Audits, practical simulation-based training, feedback by specialist nurses.* | *34.5% reduction in maintenance deficiencies; increased nurse confidence (p=0.001).* |
| ***Ballard et al. (2007)*** | *USA* | *Pressure Injury* | *Multifaceted* | *Weekly audits by specialized team; educational rounds on PI prevention and management; systematic use of Braden scale.* | *Consistent reduction in hospital-acquired pressure ulcer rates over final quarters.* |
| ***Barakat-Johnson et al. (2019)*** | *Australia* | *Pressure Injury* | *Multifaceted* | *Monthly feedback; bedside education; international partnerships for best practice exchange and strategic adjustments.* | *Significant HAPI prevalence reduction: 71.6% drop; estimated annual savings of USD 837,000.* |
| ***Bargmann & Brundrett (2022)*** | *USA* | *Patient falls* | *Multifaceted* | *Updated preventive bundle with daily patient education, frequent audits, and staff incentives ("Catch ‘em Doing Good").* | *Fall rate dropped from 1.59 to 0.54 per 1,000 patient days; alarm adherence rose to 89%.* |
| ***Barker et al. (2012)*** | *Australia* | *Pressure Injury* | *Multifaceted* | *Intensive education on risk assessment using a validated checklist; regular audits.* | *Prevalence of hospital-acquired pressure ulcers dropped from 12.6% (2003) to 2.6% (2011).* |
| ***Bartolowits et al. (2023)*** | *USA* | *Pressure Injury* | *Multifaceted* | *Monthly collaborative meetings using Lean Six Sigma methodology.* | *45% reduction in hospital-acquired injuries; increased engagement of care staff.* |
| ***Bjorklund-Lima (2022)*** | *Brazil* | *Perioperative safety* | *Multimodal* | *Hands-on training and simulation in surgical positioning, review of institutional protocols, and use of the ELPO scale.* | *96.3% of participants rated the training as "very good"; improved awareness regarding prevention of positioning injuries.* |
| ***Blanco-Mavillard (2023)*** | *Spain* | *Peripheral and central intravenous catheter-related complications* | *Multifaceted* | *Education by local facilitators, visual inspections, removal of unnecessary catheters.* | *Significant PIVC failure reduction; savings of €19.50 per catheter inserted.* |
| ***Blondal et al. (2016)*** | *Iceland* | *Catheter-Related Complications* | *Educational* | *Educational sessions by specialists (CNS and urotherapist), checklist for consistency, daily data collection, and pilot test adjustments.* | *Significant reduction in catheter days without proper indication; fewer patients discharged with catheters.* |
| ***Brown-Deveaux et al. (2022)*** | *United States* | *Safety during care transitions* | *Multifaceted* | *Redesign of bedside handover using the IPASS framework; education through presentations, videos, simulations, and checklists.* | *60% reduction in falls in the neurology unit; 22% increase in HCAHPS scores on nurse communication.* |
| ***Choong et al. (2022)*** | *Singapore* | *Hand Hygiene* | *Multifaceted* | *Educational sessions, teach-back method, regular meetings, and pre/post-implementation audits.* | *Increase in patients performing hand hygiene before meals (48.9% → 72.3%, p<0.05).* |
| ***Coutinho et al. (2022)*** | *Portugal* | *Perioperative safety* | *Multimodal* | *Use of JBI-GRiP tool to identify barriers, development of chemotherapy administration checklists, ongoing meetings and training.* | *Compliance in the "medication validity check" criterion increased from 0% to 64.29%; improved team coordination.* |
| ***Coyer et al. (2021)*** | *Australia* | *Pressure Injury* | *Multifaceted* | *Standardized assessment, in-service education rounds, continuous guideline review.* | *Prevalence of PIs reduced from 18.6% to 13.2%; HAPI incidence dropped from 7.7% to 3.3%.* |
| ***Day C. (2009)*** | *United States* | *Hand Hygiene* | *Multimodal* | *Education with practical demonstrations and visual tools to raise hand hygiene awareness.* | *Overall hand hygiene adherence increased from 45% to 85% in 6 months; 90% in nurses, 85% in doctors, 79% in other staff.* |
| ***DiGerolamo et al. (2021)*** | *USA* | *Patient falls* | *Educational* | *Interactive sessions based on real cases, pre/post-intervention assessments with practical application.* | *Staff knowledge increased (+63%); average fall reduction (-46%) after six months.* |
| ***Dobre et al. (2022)*** | *Romania* | *Pressure Injury* | *Multifaceted* | *Evidence-based prevention protocol, educational guides for patients and staff, pre/post-intervention audits.* | *Compliance with recommended practices reached 100%; significant reduction in PIs.* |
| ***Dykes et al. (2019)*** | *USA* | *Patient falls* | *Multifaceted* | *Fall TIPS Toolkit integrated into electronic records, personalized plans, regular audits.* | *25% reduction in Patient falls across four participating hospitals.* |
| ***Engel et al. (2023)*** | *USA* | *Peripheral and central intravenous catheter-related complications* | *Multifaceted* | *Regular audits and annual educational reinforcement sessions to sustain practice changes.* | *Average CLABSI rate dropped 22.8%; CHG bathing compliance rose to 94.1%.* |
| ***Eppling & Nickel (2023)*** | *United States* | *hospital-acquired pneumonia* | *Multifaceted* | *Change in VAP bundle policy, electronic audits, and ongoing staff training.* | *Improved adherence to bundle; cost savings over $21,000 with changes in oral hygiene kits.* |
| ***Esaka (2020)*** | *United States* | *Safety during care transitions* | *Multifaceted* | *Implementation of SBAR tool; audits and pre/post-intervention questionnaires.* | *Improved team communication perception (41.2 → 45.2); reduction in adverse events and RRT use.* |
| ***Fabbruzzo-Cota et al. (2016)*** | *Canada* | *Pressure Injury* | *Multifaceted* | *Interdisciplinary committee led by clinical experts, e-learning modules, practical sessions, quarterly audits.* | *80% reduction in hospital-acquired injuries.* |
| ***Gallo et al. (2019)*** | *USA* | *Pressure Injury* | *Multifaceted* | *Kaizen events with root cause analysis, development of the T3 program ("turn, touch, tidy"), care practice reorganization.* | *HAPIs reduced to zero for three consecutive quarters; total savings of USD 379,000.* |
| ***Godlock et al. (2016)*** | *USA* | *Patient falls* | *Multifaceted* | *Practical simulation (TeamSTEPPS), post-fall checklist, audits and huddles with staff and patients.* | *Fall rate reduced from 1.90 to 0.69 per 1,000 patient days; stabilized at 1.63 after one year.* |
| ***Gupta et al. (2021)*** | *Qatar* | *Peripheral and central intravenous Urinary catheter-associated complications* | *Multifaceted* | *Checklist integrated into electronic records, standardized kits, covert monitoring.* | *CLABSI eliminated for 757 days; compliance increased from 64% to 100%.* |
| ***Hoke & Guarracino (2016)*** | *USA* | *Patient falls* | *Multifaceted* | *Real-time feedback education, team discussions, data collection badges, and monthly audits.* | *55% reduction in fall rate; fall-related injuries reduced by 72%.* |
| ***Liang et al. (2019)*** | *Taiwan* | *Urinary catheter-associated complications* | *Multifaceted* | *Pre- and post-implementation audits, multidisciplinary CAUTI prevention conference, simulation modules, and self-learning materials.* | *Catheter removal within 24h increased from 37.5% to 78%; overall compliance with preventive practices significantly improved.* |
| ***Liu et al. (2022)*** | *China* | *Stroke-Associated Pneumonia* | *Multifaceted* | *Implementation of an evidence-based bundle, staff training, and environmental adjustments.* | *SAP rate reduced from 37.2% to 14%; improved patient albumin levels and oral hygiene.* |
| ***Lopez et al. (2004)*** | *USA* | *Peripheral and central intravenous catheter-related complications* | *Multifaceted* | *Educational workshops, documentation checklist, continuous nurse practice monitoring.* | *Significant reduction in phlebitis and infiltration incidence post-intervention (p<0.05).* |
| ***Maia et al. (2018)*** | *Brazil* | *Patient falls* | *Multifaceted* | *Institutional protocol adapted with Morse scale, staff training, educational materials for patients/families.* | *>75% compliance in most audited criteria; significant fall rate reduction over time.* |
| ***McCarty et al. (2018)*** | *USA* | *Patient falls* | *Multifaceted* | *MEDFRAT implemented in electronic records, in-person educational sessions adapted to local settings.* | *Preliminary results showed greater intervention adherence; long-term results pending.* |
| ***Melnyk et al. (2017)*** | *United States* | *Safety Culture* | *Multifaceted* | *Organizational assessment of EBP culture, strategic planning, and implementation of EBP-based changes.* | *Significant increase in belief and implementation of EBP among professionals.* |
| ***Mordiffi et al. (2016)*** | *Singapore* | *Patient falls* | *Multifaceted* | *Hendrich II model, TCAB framework, translated educational materials, monthly audits.* | *40% fall reduction after 10 months; long-term sustainable compliance.* |
| ***Morgan & Thomas (2007)*** | *USA* | *Peripheral and central intravenous catheter-related complications* | *Multifaceted* | *Insertion/maintenance bundles, daily checklist, hand hygiene training.* | *CR-BSI rate dropped from 5.2 to 2.0 per 1,000 catheter days.* |
| ***Nedelcu et al. (2022)*** | *Romania* | *Perioperative safety* | *Multimodal* | *Team communication education, use of standardized intraoperative safety checklists, audits, and organizational restructuring.* | *100% compliance in 8 criteria; significant improvements in communication and information transfer.* |
| ***Pol-Castañeda et al. (2022)*** | *Spain* | *Pressure Injury* | *Multifaceted* | *Incorporation of Advanced Practice Nurses (APHNs), training, monthly independent audits, periodic feedback.* | *Adherence to guidelines increased from 62% to 91%; reduction in catheter and PI-related adverse events.* |
|  |  |  |  |  |  |
| ***Rivieira et al. (2020)*** | *USA* | *Pressure Injury* | *Multifaceted* | *Implementation of preventive bundle (PIP), education during morning briefings led by expert nurses, daily and weekly audits.* | *HAPI index decreased from 3.4 to 0.48 in 10 months post-intervention; increased bundle adherence.* |
| ***Roe & Williams (2014)*** | *USA* | *Pressure Injury* | *Multifaceted* | *Interprofessional evidence-based policy review; Braden Scale module development; continuous metrics on guideline adherence.* | *HAPI prevalence dropped significantly (>11% to <2%).* |
| ***Roney, Whitley, Long et al. (2019)*** | *United States* | *Healthcare-Associated Infections (HAI)* | *Multifaceted* | *Implementation of MEWS-Sepsis tool, evidence-based review, critical scenario simulations, and PDSA cycles.* | *Sustained 24% reduction in sepsis mortality over 5 years.* |
| ***Schumacher et al. (2013)*** | *USA* | *Pressure Injury* | *Multifaceted* | *Development of “Trigger Tool” integrated into electronic records; specialized nursing consultations; educational rounds.* | *NICU PI prevalence remained low: <0.01 per thousand patient days after protocol implementation.* |
| ***Spano-Szekely et al. (2019)*** | *USA* | *Patient falls* | *Multifaceted* | *BMAT tool for mobility assessment, video monitoring, monthly interprofessional post-fall review meetings.* | *54% reduction in falls; estimated annual savings of USD 84,000 due to reduced sitter use.* |
| ***Szymaniak (2013)*** | *Australia* | *Patient falls* | *Multifaceted* | *ARST, FRAT and Safe Steps Chart tools; bed exit alarms and online training for clinical staff.* | *Fall rate reduced from 26 to 19 during implementation; >95% compliance.* |
| ***Taylor (2015)*** | *USA* | *Peripheral and central intravenous catheter-related complications* | *Multifaceted* | *Educational sessions, preventive bundle, continuous monitoring with daily safety report review.* | *Infiltration rate dropped from 20 to 8.93 per 1,000 patient days post-intervention.* |
| ***Warren et al. (2019)*** | *United States* | *hospital-acquired pneumonia* | *Educational* | *Development of oral care protocol, including patient education and provision of oral hygiene kits.* | *37% reduction in NV-HAP; estimated prevention of 16 deaths and savings of $1.04 million over 7 months.* |
| ***Wright et al. (2014)*** | *Australia* | *Patient falls* | *Multifaceted* | *Continuous education, regular audits with feedback.* | *97% compliance achieved; significant improvements in fall prevention.* |

*Table 2. Summary of included studies: patient safety events, KNOWLEDGE TRANSLATION strategy complexity, core components, and outcomes (Full version available in Supplementary File 2: PI: pressure injury; HAPI: hospital-acquired pressure injury; CLABSI: central line-associated bloodstream infection; PIVC: peripheral intravenous catheter; CR-BSI: catheter-related bloodstream infection; NV-HAP: non-ventilator hospital-acquired pneumonia; VAP: ventilator-associated pneumonia; SAP: stroke-associated pneumonia; CAUTI: catheter-associated urinary tract infection; CHG: chlorhexidine gluconate; NICU: neonatal intensive care unit; T3: turn, touch, tidy; TeamSTEPPS: team strategies and tools to enhance performance and patient safety; BMAT: bedside mobility assessment tool; SBAR: situation, background, assessment, recommendation; IPASS: illness severity, patient summary, action list, situation awareness, synthesis; MEWS: modified early warning score; PDSA: plan-do-study-act; SOP: standard operating procedure; EBP: evidence-based practice; CNS: clinical nurse specialist; ELPO: risk assessment scale for pressure injuries in surgical positioning.*

*Type of patient safety event targeted (categorized based on international literature and classifications from the International Classification for Patient Safety – WHO ICPS – and the Agency for Healthcare Research and Quality – AHRQ).*
